# Molecular Epidemiology and Antimicrobial Resistance of Methicillin-Resistant *Staphylococcus aureus* Bloodstream Isolates in Taiwan, 2010

**DOI:** 10.1371/journal.pone.0101184

**Published:** 2014-06-26

**Authors:** Chih-Jung Chen, Yhu-Chering Huang, Lin-Hui Su, Tsu-Lan Wu, Shu-Huan Huang, Chun-Chih Chien, Po-Yen Chen, Min-Chi Lu, Wen-Chien Ko

**Affiliations:** 1 Division of Pediatric Infectious Diseases, Chang Gung Memorial Hospital at Linkou, Taoyuan, Taiwan; 2 College of Medicine, Chang Gung University, Taoyuan, Taiwan; 3 Department of Laboratory Medicine, Chang Gung Memorial Hospital at Linkou, Taoyuan, Taiwan; 4 Department of Laboratory Medicine, Chang Gung Memorial Hospital at Keelung, Keelung, Taiwan; 5 Department of Laboratory Medicine, Chang Gung Memorial Hospital at Kaohsiung, Kaohsiung, Taiwan; 6 Department of Pediatrics, Taichung Veterans General Hospital, Taichung, Taiwan; 7 Department of Medicine, Chung Shan Medical University Hospital, Taichung, Taiwan; 8 Department of Medicine, National Cheng Kung University Hospital, Tainan, Taiwan; University of Mississippi Medical Center, United States of America

## Abstract

The information of molecular characteristics and antimicrobial susceptibility pattern of methicillin-resistant *Staphylococcus aureus* (MRSA) is essential for control and treatment of diseases caused by this medically important pathogen. A total of 577 clinical MRSA bloodstream isolates from six major hospitals in Taiwan were determined for molecular types, carriage of Panton-Valentine leukocidin (PVL) and *sasX* genes and susceptibilities to 9 non-beta-lactam antimicrobial agents. A total of 17 genotypes were identified in 577 strains by pulsotyping. Five major pulsotypes, which included type A (26.2%, belonging to sequence type (ST) 239, carrying type III staphylococcal chromosomal cassette *mec* (SCC*mec*), type F (18.9%, ST5-SCC*mec*II), type C (18.5%, ST59-SCC*mec*IV), type B (12.0%, ST239-SCC*mec*III) and type D (10.9%, ST59-SCC*mec*V_T_/IV), prevailed in each of the six sampled hospitals. PVL and *sasX* genes were respectively carried by ST59-type D strains and ST239 strains with high frequencies (93.7% and 99.1%, respectively) but rarely detected in strains of other genotypes. Isolates of different genotypes and from different hospitals exhibited distinct antibiograms. Multi-resistance to ≥3 non-beta-lactams was more common in ST239 isolates (100%) than in ST5 isolates (97.2%, *P* = 0.0347) and ST59 isolates (8.2%, *P*<0.0001). Multivariate analysis further indicated that the genotype, but not the hospital, was an independent factor associated with muti-resistance of the MRSA strains. In conclusion, five common MRSA clones with distinct antibiograms prevailed in the major hospitals in Taiwan in 2010. The antimicrobial susceptibility pattern of invasive MRSA was mainly determined by the clonal distribution.

## Introduction

Methicillin-resistant *Staphylococcus aureus* (MRSA) was first reported in the United Kingdom in 1961 soon after the introduction of methicillin [1]. Over the next 10 years, increasing numbers of isolates and outbreaks of the “archaic” 1960s clone.

were reported mainly in European countries [2]. After a decline in the 1970s, new epidemic strains that differed from the original MRSAs emerged in Australia, the United States and the Irish Republic in the late 1970s and early 1980s and have now prevailed in most of the hospitals in industrialised countries worldwide [3]–[6]. The evolution of MRSA has been further advanced over the past decade when the emergence of community-associated (CA)-MRSA strains occurred [7], [8]. The emerging community clones of MRSA were endemic in specific regions and spread between countries and continents. Evidence further indicated the entry of the CA-MRSA strains into healthcare facilities as a prevalent nosocomial pathogen [9], [10].

In Taiwan, MRSA was first documented in the early 1980s [11]. The incidence of nosocomial MRSA infections increased remarkably in the 1990s [12]. In the early 2000s, MRSA accounted for 53–83% of all *S. aureus* clinical isolates in most major hospitals, and most clinical isolates shared common molecular characteristics, which included a particular pulsotype with sequence types (ST) 239 or 241 and inclusion of the type III staphylococcal chromosomal cassette *mec* (SCC*mec*) [13], [14]. Since the first report of CA-MRSA in 2002, the rate of MRSA among CA *S. aureus* infections increased markedly and rapidly, particularly in children [8], [15]. For children, the rate of CA staphylococcal infections that were attributable to MRSA increased significantly from 9.8% in 1999–2000 to 56% in 2004–2005 [15], [16]. Because MRSA may evolve with time and there has been no island-wide report addressing this issue in the past decade, we conducted this study that included 577 clinical bloodstream isolates from six major hospitals in 2010 to evaluate the impact of CA-MRSA and other global pandemic nosocomial clones on the change of molecular epidemiology and antimicrobial susceptibility of MRSA in Taiwan.

## Materials and Methods

### Ethics statement

The study was approved by the institute review boards from Chang Gung Memorial Hospital, which allowed the phenotypic and genotypic characterizations of the MRSA isolates. A waiver of consent was granted given the retrospective nature of the project. The project was not involved in the collection and analysis of the demographics and clinical information of any patient.

### MRSA isolates

One hundred MRSA bloodstream isolates, respectively, were collected from each of the six participating hospitals in 2010 in Taiwan. The MRSA isolates were selected from the last bloodstream isolate in 2010 in each hospital and then consecutively isolated backward up to 100 isolates. No duplicate isolates from a single patient were included. All six hospitals were tertiary care teaching hospitals. Hospitals I and II were located in northern Taiwan, Hospitals III and IV were located in central Taiwan, and Hospitals V and VI were located in southern Taiwan. Identification of *S. aureus* was confirmed by coagulase testing, and oxacillin susceptibility was assessed by the disc diffusion method according to the Clinical and Laboratory Standard Institute 2011 guideline in the central laboratory at Chang Gung Memorial Hospital at Linkou.

### Antimicrobial susceptibility tests

The antimicrobial susceptibility of all MRSA isolates to 10 antibiotics, including oxacillin, trimethoprim/sulfamethoxazole (SXT), ciprofloxacin, clindamycin, erythromycin, doxycycline, fusidic acid, vancomycin, teicoplanin and linezolid, was tested using the disk-diffusion method in accordance with the 2011 Guideline of Clinical and Laboratory Standard Institutes.

### Molecular characterisations

Pulsed-field gel electrophoresis (PFGE) with *Sma*I digestion was performed according to the procedures described previously [13]. The genotypes that were designated were in line with a previous nationwide survey and were listed in alphabetical order [13]. PFGE patterns with fewer than 4-band differences from an existing genotype were defined as subtypes of that genotype.

SCC*mec* typing was determined by a multiplex polymerase chain reaction (PCR) strategy that was described previously [17]; and if this method was not successful in identifying a type, another multiplex PCR strategy was used [18]. The identification of SCC*mec* type V_T_ was confirmed with a method described by Boyle-Vavra et al [19]. Control strains for SCC*mec* types I, II, III and IVa were kindly provided by Dr Keiichi Hiramatsu and were as follows: type I, NCTC10442; type II, N315; type III, 85/2082; and type IVa, JCSC4744. SCC*mec* typing for type V_T_ was determined using a particular primer that was described elsewhere and the strain TSGH-17, which was used as a control [19]. The presence of Panton-Valentine leukocidin (PVL) genes was determined by a PCR strategy that was described previously [16]. Some isolates of representative PFGE patterns were subjected to multilocus sequence typing (MLST). The allelic profiles were assigned through comparison of the sequences at each locus with those of the known alleles in the *S. aureus* MLST database and were defined as sequence types (STs) accordingly. Protein A gene (*spa*) typing was performed for selected MRSA isolates. The polymorphic X region of spa was amplified by PCR using the primers 1095F (5′-AGACGATCCTTCGGTGAGC-3′) and 1517R (5′-GCTTTTGCAATGTCATTTACTG-3′) [20]. PCR products were sequenced, and spa types were assigned by analysis of the nucleotide sequences using BioNumerics version 6.5 (Applied Maths NV). The procedure for the detection of the *sasX* gene followed that described by Li et al, and the primers that were used included *sasX*-f agaattagaagtacgtctaaatgc and *sasX*-r gctgattatgtaaatgactcaaatg [21].

### Statistics

A comparison of incidences of multiple drug resistance between different pulsotypes was performed using Fisher’s exact test, while differences in numbers of drugs to which MRSA were resistant to between the sampled hospitals and clonalities of strains were tested by ANOVA method. Multiple logistic regression analysis was applied to simultaneously evaluate the hospital and clonality factors associated with multiple drug resistance. Statistical significance was defined as a *P* value of <0.05 in these tests. The data were analyzed with SAS software version 9.1 (SAS Institute Inc., Cary, NC).

## Results

Of the 600 isolates that were collected, 23 isolates from five hospitals were found to be not MRSA after re-identification at the central laboratory and were excluded. A total of 577 MRSA isolates were included in this study (Table S1). By pulsed-field gel electrophoresis (PFGE) with *Sma*I digestion, 17 genotypes with 154 subtypes were identified. The number of genotypes ranged from 8 to 11 from each single hospital. No single PFGE type accounted for greater than 30% of the 577 isolates. The most prevalent genotype was type A (26.2%), which was followed by type F (18.9%), type C (18.5%), type B (12.0%) and type D (10.9%). These five types prevailed in each of the 6 hospitals. Type BM was a relatively minor genotype (5.9%) that was more frequently identified only in hospital IV (10.3%). The detailed distribution of PFGE types of the isolates from each hospital is shown in Table 1.

**Table 1:** Resolution and distribution of PFGE patterns of 577 MRSA blood isolates from 6 major hospitals in Taiwan, 2010.

Hospital	IsolateNo.	TypeNo.	Type A		Type B		Type C		Type D		Type F		Type BM		Other Types	
			SubtypeNo.*	Isolate No.(%)	SubtypeNo.*	Isolate No. (%)	SubtypeNo.*	Isolate No.(%)	SubtypeNo.*	IsolateNo. (%)	SubtypeNo.*	IsolateNo.(%)	SubtypeNo.*	IsolateNo. (%)	Type No.	Isolate No.(%)
I	100	11	12	26 (26)	7	10 (10)	13	22 (22)	5	7 (7)	12	19 (19)	2	5 (5)	5	11 (11)
II	97	11	6	10 (10.3)	4	19 (19.6)	12	19 (19.6)	5	13 (13.4)	11	20 (20.6)	3	3 (3.1)	5	13* (13.4)
III	97	10	15	42 (43.3)	3	3 (3.1)	10	14 (14.4)	7	7 (7.2)	13	22 (22.7)	4	5 (5.2)	4	4 (4.1)
IV	97	11	9	27 (27.8)	2	4 (4.1)	8	13 (13.4)	6	13 (13.4)	16	23 (23.7)	5	10 (10.3)	5	7 (7.2)
V	89	8	7	29 (32.6)	6	14 (15.7)	9	9 (10.1)	5	10 (11.2)	8	17 (19.1)	5	5 (5.6)	2	5 (5.6)
VI	97	9	9	18 (18.6)	8	19 (19.6)	17	30 (30.9)	8	13 (13.4)	5	7 (7.2)	3	6 (6.2)	3	4 (4.1)
Total	577	17	30	152 (26.3)	15	69 (12.0)	34	107 (18.5)	22	63 (10.9)	32	108 (18.7)	10	34 (5.9)	11	44 (7.6)

*Subtype No. indicates the numbers of subtype within each major PFGE types. For instance, 12 subtypes of PFGE type A were identified in Hospital I.

**Figure 1:**
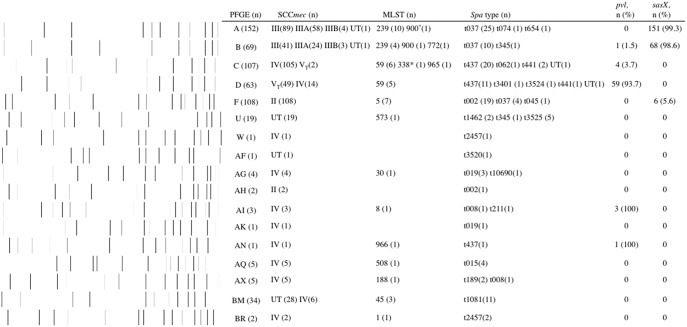
Molecular typing and carriage of virulence genes among 577 methicillin-resistant *Staphylococcus aureus* blood isolates in six major hospitals of Taiwan, 2010 stratified by pulsed-field gel electrophoresis (PFGE) types. *ST900 is a single locus variant (SLV) of ST239; ST338 is a SLV of ST59. Abbreviations: UT, untypeable; PFGE, pulsed-field gel electrophoresis; MLST, multilocus sequence type; *pvl*, Panton-Valentine leukocidin genes; *sasX*, SasX gene.

Further molecular characterisations disclosed that isolates of the six PFGE types could be clustered into four distinct genetic lineages. Isolates of type A and B were of the ST239 lineage and carried the type III or III variants SCC*mec,* whereas isolates of type C and D were of the ST59 lineage and carried a type IV or V_T_ SCC*mec*. Isolates of type F were of the ST5 lineage and carried a type II SCC*mec*. Isolates of type BM belonged to ST45. A majority (82.4%, 28/34) of the type BM isolates carried a not-yet-defined SCC*mec* element. PVL genes were harboured by 93.7% of PFGE type D isolates but were rarely detected in isolates of other genotypes. The *sasX* gene was identified in isolates of the ST239 lineage with extremely high incidence (99.1%) but was rarely identified in isolates of other genetic backgrounds (Figure 1). PFGE type F appeared to be an exception, and 6 (5.6%) isolates of this pulsotype were positive for the *sasX* gene.

All of the MRSA isolates were susceptible to vancomycin and teicoplanin. The detailed antibiograms of the isolates to the other 7 non-beta-lactam antibiotics stratified by the PFGE types are shown in Table 2. Isolates of different genetic lineages exhibited distinct patterns of antibiotic susceptibilities. All of the ST239 strains were of multiple drug resistance (resistant to ≥3 non-beta-lactams) with a median number of 5 drug resistances in isolates of type A (range, 3–7 drugs) and type B (range, 3–6 drugs) (Table 2). The multi-resistance was significantly less common in type C isolates (8.4%, *P*<0.0001), type D isolates (7.9%, *P*<0.0001), type F isolates (97.2%, *P* = 0.0347), type BM isolates (35.3% *P*<0.0001) and other isolates (31.8%, P<0.0001) compared to the ST239 isolates (all by Fisher’s exact test). Type F isolates were frequently resistant to clindamycin (98.2%), erythromycin (100%) and ciprofloxacin (97.2%) whereas type C and type D isolates were generally resistant to clindamycin (91.6% and 87.3%, respectively) and erythromycin (91.6% and 92.1%, respectively).

**Table 2:** Antibiotic non-susceptibility rate* of 577 methicillin-resistant *Staphylococcus aureus* bloodstream isolates from six major hospitals in Taiwan in 2010 stratified by pulsed-field gel electrophoresis (PFGE) types.

PFGE types(isolate No.)	%								No. of drug non-susceptibility, median (range)
	CL	EM	FA	SXT	DOX	CIP	LZD	Multiresistance to ≥3 drugs	
A (n = 152)	93.4	100	28.5	98.7	91.4	99.3	0.7	100.0	5 (3–7)
B (n = 69)	97.1	100	10.1	97.1	69.6	100.0	0	100.0	5 (3–6)
F (n = 108)	98.2	100	12.8	10.1	5.5	97.2	0	97.2	3 (2–5)
C (n = 107)	91.6	91.6	1.0	2.9	1.0	4.7	0	8.4	2 (0–4)
D (n = 63)	87.3	92.1	1.6	3.2	0	11.1	0	7.9	2 (0–4)
BM (n = 34)	12	32.4	29.4	0	67.6	79.4	0	35.3	2 (0–5)
Others (n = 44)	34.1	61.4	0	4.6	38.6	43.2	0	31.8	2 (0–4)
Total (n = 577)	84.6	90.6	13.0	40.7	40.4	66.2	0.2	63.4	3 (0–7)

CL, clindamycin; EM, erythromycin; FA, fusidic acid; SXT, trimethoprim/sulfamethoxazole; DOX, doxycycline; CIP, ciprofloxacin; LZD, linezolid;

*Non-susceptibility included intermediate resistance and resistance by disc diffusion method. All 577 isolates were susceptible to vancomycin and teicoplanin.

The susceptibility pattern also differed significantly among the 6 sampled hospitals, with the highest multi-resistant rate in hospital V (75.3%, *P* = 0.0087 by chi-square test) and the greatest median number of drug non-susceptibility in hospital III (4 drugs, range 0–7, *P* = 0.0119 by ANOVA test). Multivariate analysis evaluating the pulsotypes and sampled hospitals in predicting multi-resistance indicated that the multi-resistance was not associated with hospitals (*P*>0.05 for hospital II to IV, hospital I as referent) but significantly associated with isolates of pulsotype A (adjusted odds ratio [aOR] 163.978, 95% confidence interval [CI] 73.7764–364.525, *P*<0.0001), pulsotype B (aOR 50.689, 95% CI 22.616–113.606, *P*<0.0001), pulsotype C (aOR 0.027, 95% CI 0.013–0.005, *P*<0.0001), pulsotype D (aOR 0.025, 95% CI 0.012–0.054, *P*<0.0001), pulsotype BM (0.052, 95% CI 0.022–0.125, *P*<0.0001) and other pulsotypes (aOR 0.022, 95% CI 0.009–0.051, *P*<0.0001) when the pulsotype F was set as a referent.

## Discussion

### Changed molecular epidemiology of MRSA

**Figure 2:**
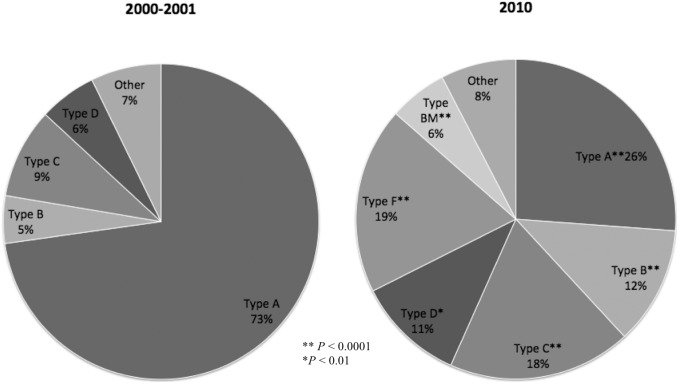
Comparison of genotype distribution among the clinical MRSA isolates in two national surveys in 2000–2001 and in 2010 in Taiwan.

A national survey of the molecular epidemiology of MRSA in 2000–2001 disclosed a single clone (PFGE type A, ST239) that prevailed in the major hospitals in Taiwan [13]. None of the other minor clones accounted for more than 10% of the sampled isolates at that time (Figure 2). Using a similar study design, results from the current study demonstrated a completely different molecular epidemiology of MRSA in Taiwan 10 years later. The dominance of the PFGE type A clone was challenged, and the proportion of type A strains declined markedly from 73% to 26.2% (*p*<0.0001, Figure 2). The waning dominance of PFGE type A was accompanied by significant increases of pulsotypes B (ST239), C and D (ST59) and the emergence of pulsotype F (ST5). The predominance of the MRSA isolates that were represented by the five major genotypes were not confined to particular sites, but to all six sampled hospitals, indicating an island-wide dissemination of the five major MRSA clones in Taiwan during 2000 and 2010.

### ST5 strains

PFGE type F (ST5) with type II SCC*mec* was identified in a few isolates in the 2000–2001 survey, but the incidence reached to approximately 20% in 2010. The predominance of the ST5 strains in Taiwan was also demonstrated in another surveillance study in 2008, which disclosed a high rate (42%) of ST5 strains among 38 nasal MRSA isolates from patients hospitalised in intensive care units of a medical center (Hospital I in the present study) [22]. A longitudinal study characterising the MRSA blood isolates that was collected during 1995 and 2006 in northern Taiwan further demonstrated the emergence of ST5 strains in 1998 with an increasing incidence during the study period [23]. ST5 with SCC*mec* II has been identified as a pandemic, nosocomial MRSA clone, namely the New York/Japan clone that circulated in the US, Canada, Japan and several regions in Europe before 2000 [3]. The rapid increase of the ST5 clone in Taiwan indicated a selective advantage of the strains in the hospital environments of this island since its emergence in the late 1990s.

### ST59 strains

CA-MRSA strains emerged in the late 1990s and spread throughout the world within a few years [7], [24]. Over the past decade, we also encountered an epidemic of CA-MRSA in Taiwan. Molecular epidemiology studies had clearly demonstrated that two clones, which were characterised as PFGE type C/SCC*mec* IV/PVL– and PFGE type D/SCC*mec* VT/PVL+ and belonged to the ST59 lineage, predominated the Taiwanese CA-MRSA strains [8]. Entry of CA-MRSA strains into hospitals as nosocomial strains had been documented [10], [25]. Although the information regarding the acquisition of MRSA was not available in the current study, we believed a majority of the blood isolates were of nosocomial origin because bacteraemia was an infrequent entity in the spectrum of CA-MRSA diseases [26], [27]. The finding that the proportion of ST59 strains in MRSA isolates increased from 15% in 2000–2001 to 30% in 2010 further suggested a well-adaptation of the ST59 clone to the hospital environments. ST59 was not only a prevalent CA-MRSA clone in Asian-Pacific region but appeared to be a successful nosocomial clone that was capable of causing invasive infections in the hospitals of Taiwan.

### ST45 strains

PFGE-type BM strains are of the ST45 lineage, which is related to the Berlin strain of MRSA [28]. The clone was firstly reported in central Europe during the 1990s and subsequently in several regions of the world, including North America, Israel and Eastern Asia [29]–[31]. Most of the reported ST45 strains carried a type IV or V SCC*mec* element. However, a majority of the ST45 strains that were identified in the current study harboured an undetermined SCC*mec* type, which suggested that the ST45 strains might have undergone significant genetic evolution involving the SCC*mec* element before spreading in the hospitals of Taiwan.

### *sasX* gene and ST239 strains

The *sasX* gene is a novel staphylococcal gene encoding a surface-anchored protein that was increasingly identified in the MRSA strains of the ST239 background during 2003 and 2011 in China [21]. It has been demonstrated that the SasX protein can promote nasal colonisation and enhance the virulence of *S. aureus.* The molecule was further proposed as the crucial factor contributing to the MRSA epidemic in Asia [21], [32]. However, this speculation was not supported by our molecular epidemiology studies. One study that characterised the molecular features of blood isolates collected during 2000–2001 disclosed that 99.0% of the 99 ST239 strains carried the *sasX* gene but this gene was not detected among non-ST239 strains (YC Huang, unpublished data). Results from the current study further consolidated the previous finding that *sasX* gene was harboured by nearly all of the strains (99.1%) of the ST239 lineage but was rarely identified in non-ST239 strains, including ST5, ST59 and ST45. The proportion of strains of the ST239 lineage among the MRSA isolates declined significantly from 78% in 2000 to 38% in 2010. The strains lacking *sasX* gene emerged and accounted for an increasing proportion of the invasive MRSA isolates in the past decade in Taiwan. Taken together, the data indicated that the *sasX* gene was a previously existing genetic marker of the ST239 strains in Taiwan before 2000. MRSA strains carrying the *sasX* gene lost their competitive advantage to other emerging strains absent for this marker. Epidemiological observations questioned the significance of this genetic marker in the MRSA epidemic. However, more molecular epidemiology studies addressing the prevalence of the *sasX* gene in MRSA strains of different clones and from distinct geographic regions are needed to clarify this issue.

### Potential impact of MRSA clone replacement

The impact of the replacement of hospital-adapted MRSA strains (PFGE type A-ST239) with the widely disseminated, community MRSA strains (ST59 and ST45) remains unclear. Current evidence suggested that community strains with or without PVL genes were more virulent than the global pandemic, nosocomial MRSA strains, although the greater virulence appeared to be confined to skin and soft tissue infections in clinical studies or experimental animal models [9], [33], [34]. Nevertheless, data from the current study clearly demonstrated that the community strains were capable of causing invasive infections and accounted for an increasing proportion of bloodstream infections in hospital settings. Whether the severity of the invasive infections or patient outcomes were distinct for strains of distinct genetic background requires further study.

It was very likely that the changes in the molecular epidemiology of MRSA resulted in a general reduction of drug resistance in MRSA blood isolates. The PFGE type A strains were associated with the highest incidence of multi-resistance, whereas the other emerging genotypes (PFGE type C, D and BM) were frequently susceptible to various antibiotics (Table 2). The growth advantage of the susceptible clone may be a plausible explanation for this phenomenon. The clone replacement with multidrug-susceptible MRSA strains could allow for a more flexible choice of antibiotics in the management of MRSA infections. For instance, clindamycin and trimethoprim-sulfamethoxazole, to which the emerging MRSA clones are more frequently susceptible, are potential therapeutic agents for treating invasive MRSA infections [35]–[37]. A reduction of the selective pressure of vancomycin may be anticipated if antibiotics other than glycopeptides can be used for the management of MRSA infections.

### Conclusion

In conclusion, when compared to the data in 2000–2001, we observed a completely different picture of the molecular epidemiology of MRSA in the major hospitals of Taiwan in 2010. The predominance of the PFGE type A-ST239-SCC*mec* III/III variant clone waned, and this was accompanied by the emergence and island-wide dissemination of another pandemic nosocomial clone, ST5-SCC*mec*II, a prevalent Asian community clone, ST59-SCC*mec* IV/V_T_, and a minor clone, ST45, with undetermined SCC*mec* types. The clone replacement resulted in a general reduction in drug resistance and may impact the management of invasive MRSA infections in Taiwan.

## Supporting Information

Table S1 The detailed information of 577 methicillin-resistant *Staphylococcus aureus* blood isolates collected from six major hospitals in Taiwan in 2010.
(XLSX)
